# The dynamics of risk perceptions and precautionary behavior in response to 2009 (H1N1) pandemic influenza

**DOI:** 10.1186/1471-2334-10-296

**Published:** 2010-10-14

**Authors:** Yoko Ibuka, Gretchen B Chapman, Lauren A Meyers, Meng Li, Alison P Galvani

**Affiliations:** 1 Department of Epidemiology and Public Health, Yale School of Medicine, 60 College Street, New Haven, CT 06520, USA; 2 Department of Psychology, Rutgers University, 152 Frelinghuysen Road, Piscataway, NJ 08854, USA; 3 Section of Integrative Biology, The University of Texas at Austin, Austin, TX 78712, USA; 4 Santa Fe Institute, 1399 Hyde Park Road, Santa Fe, NM 87501, USA

## Abstract

**Background:**

The trajectory of an infectious disease outbreak is affected by the behavior of individuals, and the behavior is often related to individuals' risk perception. We assessed temporal changes and geographical differences in risk perceptions and precautionary behaviors in response to H1N1 influenza.

**Methods:**

1,290 US adults completed an online survey on risk perceptions, interests in pharmaceutical interventions (preventive intervention and curative intervention), and engagement in precautionary activities (information seeking activities and taking quarantine measures) in response to H1N1 influenza between April 28 and May 27 2009. Associations of risk perceptions and precautionary behaviors with respondents' sex, age, and household size were analyzed. Linear and quadratic time trends were assessed by regression analyses. Geographic differences in risk perception and precautionary behaviors were evaluated. Predictors of willingness to take pharmaceutical intervention were analyzed.

**Results:**

Respondents from larger households reported stronger interest in taking medications and engaged in more precautionary activities, as would be normatively predicted. Perceived risk increased over time, whereas interest in pharmaceutical preventive interventions and the engagement in some precautionary activities decreased over time. Respondents who live in states with higher H1N1 incidence per population perceived a higher likelihood of influenza infection, but did not express greater interests in pharmaceutical interventions, nor did they engage in a higher degree of precautionary activities. Perceived likelihood of influenza infection, willingness to take medications and engagement in information seeking activities were higher for women than men.

**Conclusions:**

Perceived risk of infection and precautionary behavior can be dynamic in time, and differ by demographic characteristics and geographical locations. These patterns will likely influence the effectiveness of disease control measures.

## Background

The medical outcomes of an infectious disease outbreak are affected by the behavior of individuals. Individuals who vaccinate, take anti-viral medications or stay home from work reduce not only their own risk of infection, but also those of others in the population. The dynamic nature of infectious disease transmission means that behavior by a modest number of individuals can have a significant impact on the trajectory of an outbreak [1]. Understanding individuals' behavior and its relation to their perceived risk is therefore important in terms of effective control of an infectious disease outbreak [2].

As the classic Health Belief Model and other health psychology models indicate, risk perceptions have been viewed as one of key drivers of health behaviors [3-7]. As an example, a meta-analysis showed that perceived risk likelihood and severity judgements predict vaccination behavior and that the relationship was stronger with better measures of perceived risk [4]. Precautionary behavior may also be influenced by the perceived costs and benefits of such behavior [8,9], and the perceived impact of an individual's behavior on other individual's health outcomes [10]. Consequently, when predictors of precautionary behaviors, such as perceived risks and benefits change over time, we might expect the precautionary behavior to follow suite. An unexpected outbreak of infectious disease provides an opportunity to examine how new information affects risk perceptions and, hence, how changes in risk perceptions influence behavior. When the pandemic outbreak of H1N1 influenza started in Mexico City in April 2009, the massive media coverage in the early days of the outbreak coupled with global disease transmission instigated widespread fear initially [11,12]. However, media attention declined gradually in the U.S [13]. Understanding the factors that drive individual behaviors in dynamic social contexts has both theoretical and practical implications.

In addition to the temporal change, the incidence of H1N1influenza in the US shows substantial geographical variation [14]. Consequently, objective risk of infection varies with geography. The outbreak therefore afforded the opportunity to examine whether perceived risk tracks objective risk. We examined the geographical differences in risk perceptions and precautionary behaviors.

Recent studies examined risk perceptions, emotions, and precautionary behaviors during the early stage of the 2009 (H1N1) influenza pandemic in Britain, Hong Kong, Australia, Malaysia and Europe, and the USA [13,15-21]. These studies found that precautionary behaviors were associated with anxiety about H1N1 influenza [13,16,17,19-21], risk perceptions [13,15,16,18-21], perceived efficacy of the precautionary behaviors [16,20], and prior experience of influenza vaccination [15,18,21].

Our study focuses on the temporal and geographic dynamics of risk perceptions and precautionary behaviors. We examined how risk perception, willingness to take pharmaceutical interventions, and engagement in precautionary activities changed over time and differed by geographical risk status in each state. In addition, we compared survey responses to the number of H1N1-related articles published in newspapers to provide a preliminary look at the relationship between precautionary behaviors and media attention. Our measures of risk perception included perceived likelihood of infection and perceived severity [4].

## Methods

### Data collection

Potential study participants were contacted via email by a professional survey firm (Survey Sampling International, Shelton, CT) and directed to the survey web site. The survey company had a list of individuals that had consented to receive invitations for surveys although they were free to decline any invitation they received. The first invitations were sent on April 28 2009. Each survey day, recruitment started at noon and lasted until our daily quota was achieved. We collected approximately 500 responses as the basis of the study on the first two survey days. We then set the quota to be 50 between April 30 and May 12, and 100 on May 19 and 26, collecting a cross-sectional sample of the US population on each day (Additional files 1 &2). A total of 45,786 potential participants were invited; 1,370 initiated the questionnaire, and 1,290 provided complete responses and were included in the analysis. Non-responders included people who declined to participate and those who were turned away after the daily quota had been reached. Analysis of non-respondents' demographic characteristics obtained from the survey company indicated that there was substantial difference in response rate by sex as well as by age: women were more likely to respond than men, and the response rate sharply increased with age, ranging from 1.0% for those aged 18 to 29 to 12% for those aged 65 or over.

The survey company issued invitations in such a way that the age and gender distribution of participants would approximate that of the US adult population on each day. The survey procedure did not allow us to identify the exact proportion of those who did not participate and of those who were turned away after the daily quota was reached.

Our research conformed to the Helsinki Declaration outlining the principles for medical research involving human subjects. Participants provided informed consent to participate in the study. Committees from Rutgers University institutional review board approved the research protocol.

### Survey items on risk perception and precautionary behaviors

In the first question of the survey, respondents were asked if they had known about H1N1 influenza before opening the survey, and from whom or what they first learned about the H1N1 influenza outbreak. The survey included three questions on respondent's risk perception toward H1N1 influenza, six questions on willingness to take pharmaceutical intervention, and six questions on precautionary activities (Table 1). Among the three items about risk perception, the first two questions on the likelihood of contact with H1N1 influenza were highly correlated (Cronbach's α = 0.85) and were combined to form a *perceived likelihood scale*. The third risk perception question elicited perceived death toll from H1N1 influenza, which is a measure of perceived severity.

**Table 1:** Survey items on risk perceptions and precautionary behaviors.

Questions	Choices of answers	N	Statistics
Perceived risk			

In your opinion, what is the likelihood that swine flu will reach your community?	0%, 10%, 20%, 30%, 40%, 50%, 60%, 70%, 80%, 90%, 100%	1288	37.9%* (30.2)

In your opinion, what is the likelihood that you will personally encounter somebody infected with swine flu?	0%, 10%, 20%, 30%, 40%, 50%, 60%, 70%, 80%, 90%, 100%	1247	25.6%* (24.6)

In your opinion, how many people worldwide will die from swine flu during this outbreak?	<100; 100-1,000; 10,000-100,000; 100,000-1,000,000; >1,000,000	1290	100-1,000†

Precautionary behaviors			

Willingness to take pharmaceutical interventions			

If a vaccine for swine flu became available, would you want to be vaccinated?	Yes/No	1290	57.6%‡

If a vaccine for swine flu became available, what is the maximum you would pay to become vaccinated?	$0, $20, $50, $100, $150, $200, $500, $1,000, $5,000, $10,000, $20,000	1290	20†

Antiviral medications can be taken during an outbreak to prevent infection. To be effective, they must be taken for the entire duration of the epidemic. If antiviral medications were available for swine flu, would you want to take them?	Yes/No	1290	57.1%‡

How much would you pay for antiviral medication (enough doses to last the duration of the epidemic)?	$0, $20, $50, $100, $150, $200, $500, $1,000, $5,000, $10,000, $20,000	1290	20†

Antiviral medications are also used for treating cases of flu. If you became infected with swine flu, would you seek treatment with antiviral medications?	Yes/No	1290	83.2%‡

How much would you pay for antiviral treatment if you were infected?	$0, $20, $50, $100, $150, $200, $500, $1,000, $5,000, $10,000, $20,000,	1290	20†

Engagement in precautionary activities			

For each of the following questions, please indicate whether or not you have changed your behavior in response to swine flu outbreak.			

Are you following television or radio news more closely in response to the swine flu outbreak?	Yes/No	1218	51.2%‡

Have you searched the internet for additional information on the swine flu outbreak?	Yes/No	1220	28.7%‡

Have you cancelled or changed travel plans in response to the swine flu outbreak?	Yes/No	1219	4.4%‡

Have you or your children stayed home from school in response to the swine flu outbreak?	Yes/No	1214	3.5%‡

Have you stayed home from work in response to the swine flu outbreak?	Yes/No	1218	2.1%‡

Have you cancelled or changed social plans in response to the swine flu outbreak?	Yes/No	1219	5.1%‡

The symbols in the table show: * mean value (standard deviation); † median value; and ‡ proportion of respondents who answered yes, respectively.

The six items on willingness to accept pharmaceutical interventions included items on vaccination and antiviral medications. These six items were grouped into two sections: willingness to accept preventive interventions (vaccine and prophylactic anti-viral medication) and willingness to accept curative interventions (prophylactic anti-viral medication). Further each of the two types of interventions was grouped into two scales, a yes/no scale of whether respondents were interested in intervention, and standardised willingness to pay (WTP) scale. We created a dichotomous omnibus measure to indicate whether respondents showed interest in either of the two preventives. Two items on willingness to pay to receive preventive intervention were standardized and combined (Cronbach's α = 0.86). The willingness to pay to receive curative intervention was also standardized.

The six items about precautionary behaviors, which asked whether respondents had already engaged in various precautionary measures, were grouped into two scales: information seeking activities (2 items), and quarantine measures (4 items). We created two dichotomous measures to indicate whether respondents engaged in any of the precautionary activities in each scale. Respondents' demographic information included sex, age, zip code, and household size.

### Geographical risk status

To analyze the geographical difference in risk perception and precautionary behaviors, we used the following three measures to describe geographical risk status by state as of May 27 2009: (1) cumulative number of H1N1 confirmed cases; (2) cumulative H1N1 confirmed cases per million population; and (3) a dichotomous variable to indicate whether the state reported any confirmed deaths (Additional file 3). Both H1N1 influenza cases and population size vary across states [14]. We chose cumulative cases rather than new infection rate as an objective risk measure, based on a previous study on SARS [22]. As there were only a small number of reported deaths caused by H1N1 influenza as of the date, ranging from zero to three in Arizona and Texas, we treated the measure to describe mortality as a dichotomous variable rather than a continuous variable.

### News stories

To analyze the association of media attention with risk perception and precautionary behaviors, news stories about the influenza outbreak were tracked, using the news search function in *LexisNexis^® ^Academic *(Reed Elsevier, Amsterdam), a comprehensive database of national and regional news media [23]. US newspapers and wires were used for sources of our search, which include approximately 700 media sources in the United States. "Flu" was used as the search term. The search results were shown classified into subgroups such as newspapers, newswires and press releases, and other sources, and the number of US newspaper articles each day between April 28 and May 26 2009 was counted. We did not include news in newswires and press releases and other news sources to avoid potential double-counting of news. Search results were narrowed by region to select news only about the US. The average number of newspaper articles each day during the period was 137 (s.d.126), with a maximum of 408 on April 30 and a minimum of 22 on May 25. Television and radio broadcast transcripts were also searched with the same search condition, and the number of transcripts was found to be correlated to the number of newspaper articles during the period of our analysis (*r *= 0.91). We therefore chose to use the number of newspaper articles following a previous study on media coverage in medicine [24].

### Statistical analysis

Statistical analyses included t-test (*t*), Pearson's chi-square test (*χ*^*2*^), Pearson's correlation coefficient (*r*) and Spearman rank-order rho (*ρ*), as appropriate. Regression analysis included linear regression, logistic regression, and ordered logistic regression for continuous, dichotomous, and ordered categorical dependent variables respectively. For time series analyses, a time variable that represents the number of days from the first day of the survey and its square were included as independent variables in regression together with sex, age and household size. The geographical analysis was also conditioned by sex, age and household size. To assess the determinants of interest in pharmaceutical interventions, the four measures of pharmaceutical intervention scale were regressed on the perceived likelihood scale, predicted death toll, two dichotomous measures indicating whether seeking information activities or quarantine measures was adopted, linear time trend, sex, age, household size and the geographical risk level measured by H1N1 cases in respondent's state. Estimates for coefficient are reported as *β*. All the statistical analyses were conducted by SAS Version 9.2 (SAS Institute, Inc. Cary, NC). To link zip codes with state, zip code files were downloaded from the April 2009 version of SAS Maps online (SAS Institute, Inc. Cary, NC, 2009).

## Results

### Perceived risk and precautionary behavior by age, sex and household size

The age and gender distribution of the respondents approximated that of the US adult population (Table 2). Most respondents (95%) reported that they had heard about H1N1 influenza before opening the survey, but familiarity was positively associated with age (*r *= 0.18, *p *< 0.001).

**Table 2:** Demographic distribution of respondents.

		N	Percentage*
Total			
		1290	100
Sex			
	Men	630	49
	Women	660	51
Age†			
	18 - 29	171	13
	30 - 39	240	19
	40 - 49	274	21
	50 - 64	361	28
	65 or older	235	18
House hold size			
	1	271	21
	2	483	37
	3 or 4	392	30
	5 or more	144	11

* Percentages may not total to 100 due to rounding. †9 respondents answered an invalid number for age.

Women gave higher mean responses than men on the perceived likelihood scale (33.2% vs. 30.4%, *t*(1288) = 1.96, *p *= 0.05). In contrast, men were more pessimistic with regard to the number of deaths from H1N1 influenza, and a larger proportion of men expected over 1,000 deaths (34.3% vs.21.7%, *χ*^2 ^= 25.6, *p *< 0.001). A higher proportion of women showed interest in taking pharmaceutical interventions than men (72.6% vs. 66.7%, *χ*^2^= 5.33, *p *= 0.02 for preventive intervention; 86.7% vs. 79.5%, *χ*^2^= 11.8, *p *< 0.001 for curative intervention), and engaged in information seeking activities (61.8% vs. 49.2%, *χ*^2^= 20.8, *p *< 0.001). There was no significant sex difference in the scores of the WTP scales, or in the proportion of respondents who took quarantine measures.

There was no consistent correlation between age and the perceived likelihood scale or engagement in precautionary activities (Table 3). However, older respondents estimated a higher death toll. Scores on the willingness to pay scale for pharmaceutical interventions were negatively correlated with age. The willingness to accept pharmaceutical intervention increased with household size, as did the engagement in information seeking activities.

**Table 3:** Perceived risk and precautionary behaviors in response to H1N1 influenza by age and household size.

	Age		Household size	
	**Correlation Coefficient**	***P*-value**	**Correlation Coefficient**	***P*-value**

Risk perception				
Perceived likelihood scale	0.04	0.19	0.08	0.003
Predicted death toll	0.11	<0.001	-0.08	0.01
Willingness to accept pharmaceutical intervention				
Preventive intervention-Interest in intervention	0.02	0.38	0.06	0.02
Preventive intervention-WTP scale	-0.13	<0.001	0.12	<0.001
Curative intervention-Interest in intervention	-0.03	0.35	0.09	0.001
Curative intervention-WTP scale	-0.16	<0.001	0.12	<0.001
Engagement in precautionary activities				
Information seeking activities	0.03	0.21	0.07	0.02
Taking quarantine measures	-0.04	0.18	0.05	0.07

The table shows association between the scale of perceived risk or precautionary behaviors in response to H1N1 influenza, and age or household size. Correlation coefficient presents Spearman's rho for predicted death toll, and Pearson's correlation coefficient for other measures. WTP = willingness to pay.

### Change in perceived risk and precautionary behavior over time

**Figure 1:**
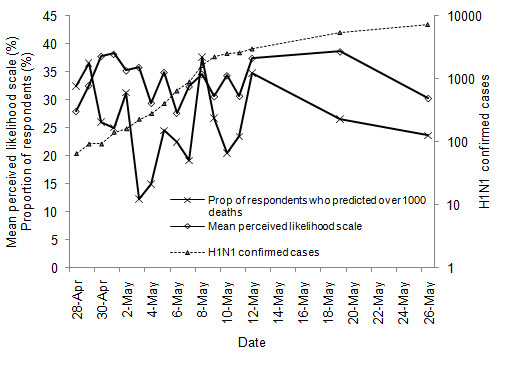
**Dynamics of H1N1-related risk perceptions and H1N1 confirmed cases, April 28 - May 26 2009**. The lines show the mean score of perceived risk scale, the proportion of respondents who predicted over 1000 deaths, and the cumulative H1N1 cases. Sources for H1N1 cases: CDC [14].

The mean perceived likelihood showed a slight increasing trend (Figure 1). Conditioned on demographic variables, regression analyses revealed a positive linear trend (*β *= 0.008, *p *= 0.004) and a negative quadratic trend (*β *= -0.0002, *p *= 0.01), indicating that the increase is steeper initially and then levels out. Predicted death toll showed a significant decrease over time (*β *= -0.06, *p *= 0.002) with a positive quadratic trend (*β *= 0.002, *p *= 0.01), indicating a more rapid decrease during the very early stage of the pandemic.

**Figure 2:**
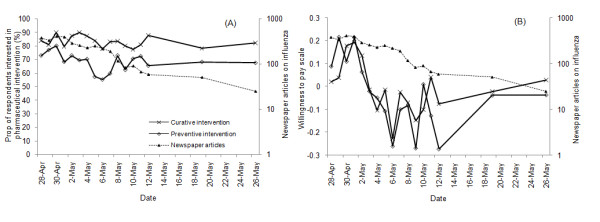
**Dynamics of willingness to accept pharmaceutical intervention and number of newspaper articles on influenza, April 28 - May 26 2009**. (A) Proportion of respondents interested in receiving pharmaceuticul interveniton and the number of newspaper articles. The lines show the proportion of those interested in preventive intervention, the proportion of those interested in curative intervention, and the number of newspaper articles. For the source of the number of newspaper articles, see text. (B) Mean scores of willingness to pay scale and the number of newspaper articles. The lines show the mean score of WTP scale for preventive intervention, the mean score of WTP scale for curative intervention, and the number of newspaper articles. For the source of the number of newspaper articles, see text.

**Figure 3:**
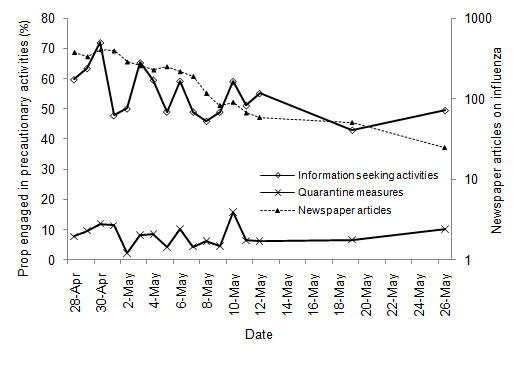
**Dynamics of engagement in precautionary activities and number of newspaper articles on influenza, April 28 - May 26 2009**. The lines show the proportion of respondents who engaged in information seeking activities, the proportion of respondents who took quarantine measures, and the number of newspaper articles. For the source of the number of newspaper articles, see text.

In contrast to the perceived likelihood, some precautionary behavior declined over time (Figures 2 &3). The proportion of respondents interested in taking preventive interventions declined over time (*β *= -0.06, *p *= 0.01). Scores on the willingness to pay scale for preventive interventions decreased over time (*β *= -0.03, *p *= 0.002) with a positive quadratic trend (*β *= 0.001, *p *= 0.01), indicating that willingness to accept preventive intervention declined over time with an initial steeper decline. The negative time trend was not observed for the two measures of curative interventions however. The probability that the respondents were engaged in information seeking activities also showed a significant negative trend over the survey period (*β *= -0.05, *p *= 0.02). This trend was not found in taking quarantine measures.

The reduction in interest regarding pharmaceutical interventions and engagement in precautionary activities may be linked to declining media attention during the survey period. Ninety two percent of the respondents reported that they first learned about the H1N1 influenza outbreak through radio (8.0%), online source (17.9%) or TV (65.9%), suggesting the importance of mass media in information collection regarding the outbreak. From April 28, the number of news articles per day shows a systematic decline that roughly parallels the decline in willingness to accept preventive interventions and information seeking activities (Figures 2 &3). Indeed, the two measures of willingness to accept preventive intervention were positively correlated with the number of H1N1 influenza articles (*r *= 0.06, *p *= 0.04 for the interest scale; *r *= 0.10, *p *< 0.001 for the WTP scale), as was engagement in information seeking activities (*r *= 0.09, *p *< 0.001). However, the incidence of H1N1 continued to rise during the period, consistent with the respondents' perceptions of increasing likelihood of contact with H1N1 influenza (Figure 1).

### Geographical difference in perceived risk and precautionary behavior

The number of confirmed influenza cases showed substantial geographical variation, with state totals ranging from none (Alaska, West Virginia and Wyoming) to 1,358 (Texas) as of May 27 (Additional file 3). We used three measures to represent geographical risk status, but our findings were mostly consistent among the three measures. Controlling for age, sex and household size, perceived global death toll was not associated with the risk measures, mirroring that the question is not geographically specific (Table 4). By contrast, responses on the perceived likelihood scale were significantly associated with geographical risk measured by H1N1 cases. Scores on the pharmaceutical intervention scales did not significantly vary with geographic risk status, however, nor did engagement in precautionary activities with the exception that taking quarantine measures was correlated with geographic risk when the later was operationalized in terms of H1N1 cases (Table 4).

**Table 4:** Perceived risk and precautionary behaviors in response to H1N1 influenza by geography.

Measures of geographical risk status	H1N1 confirmed cases		H1N1 confirmed cases per million		Confirmed deaths	
	**Coefficient estimates**	***P*-value**	**Coefficient estimates**	***P*-value**	**Coefficient estimates**	***P*-value**

Risk perception						
Perceived likelihood scale*	0.0001	<0.001	0.0006	0.002	0.03	0.08
Predicted death toll†	-0.0002	0.11	0.0001	0.95	-0.16	0.23
Willingness to accept pharmaceutical intervention						
Preventive intervention - Interest in intervention‡	-0.0001	0.44	-0.001	0.41	-0.02	0.9
Preventive intervention - WTP scale*	0	0.77	-0.0007	0.33	0.04	0.58
Curative intervention - Interest in intervention‡	-0.0002	0.26	-0.002	0.3	-0.1	0.6
Curative intervention - WTP scale*	-0.0001	0.06	-0.001	0.06	-0.04	0.59
Engagement in precautionary activities						
Information seeking activities‡	0.0001	0.51	-0.001	0.52	-0.03	0.84
Taking quarantine measures‡	0.0007	0.01	0.003	0.24	0.25	0.31

The table shows association between the scale of perceived risk or precautionary behavior in response to H1N1 influenza and geographic risk status, measured by three different indicators of risk status. The analysis was controlled for age, sex and household size. Coefficient estimates show: * linear regression coefficient; † ordered logistic regression coefficient; and ‡ logistic regression coefficient. H1N1 confirmed cases and H1N1 confirmed cases per million were indicated as continuous variables, whereas confirmed deaths were indicated as a dichotomous variable. See text and Additional file 3 for sources and details. WTP = willingness to pay.

### Predictors of pharmaceutical interventions

To examine the factors associated with willingness to accept a vaccine or antiviral pharmaceuticals, we performed regression analyses, with the four scales of willingness to accept pharmaceutical intervention as the dependent variable (Table 5). Significant predictors for all the four scales of willingness to accept pharmaceutical intervention included perceived likelihood scale and engagement in information seeking activities. Taking quarantine measures also predicted three of the four scales of willingness to accept pharmaceutical interventions. Sex was not a significant predictor any more except of the scale of interest in curative intervention, suggesting that perceived likelihood mediates the relationship between sex and willingness to receive pharmaceutical interventions. Estimated death toll and respondent's age were significant predictors of the willingness to pay scores, but not of the interest scales. Household size was a positive predictor of three scales. Overall results did not change when the two alternative measures for geographic risk level were used.

**Table 5:** Predictors of the willingness to accept pharmaceutical interventions.

	Preventive intervention				Curative intervention			
	Interest in intervention*		**Willingness to pay†**		Interest in intervention*		**Willingness to pay†**	

	**Coefficient estimate**	***P*-value**	**Coefficient estimate**	***P*-value**	**Coefficient estimate**	**P-value**	**Coefficient estimate**	***P*-value**

Perceived likelihood scale	1.83	<0.001	0.52	<0.001	1.82	<0.001	0.32	0.004
Estimated death toll	0.12	0.13	0.12	<0.001	-0.07	0.45	0.11	<0.001
Information seeking activities	0.78	<0.001	0.24	<0.001	1.03	<0.001	0.20	<0.001
Quarantine measures	0.84	0.02	0.55	<0.001	-0.29	0.42	0.39	<0.001
Household size	0.08	0.12	0.04	0.02	0.14	0.03	0.04	0.04
Sex	0.19	0.16	0.03	0.50	0.39	0.02	0.09	0.09
Age	0.005	0.28	-0.01	<0.001	-0.002	0.61	-0.01	<0.001
H1N1 cases in respondent's state	-0.0003	0.10	-0.0001	0.20	-0.0004	0.08	-0.0002	0.01
Days since the first day	-0.01	0.09	-0.01	0.08	-0.01	0.34	0.00	0.71

The table presents regression results to examine predictors of the four scales of willingness to accept pharmaceutical interventions. * shows results from logistic regressions and † shows results from linear regressions.

## Discussion

Our survey conducted at the initial stage of outbreak indicated that perceptions about the risks associated with 2009 (H1N1) pandemic influenza, as well as interest in pharmaceutical interventions and precautionary activities, showed changes over time and variations over geography and demography. Although the perceived likelihood of H1N1 infection increased over time, interest in preventive pharmaceutical interventions and engagement in information seeking activities declined. These declines were correlated with the decrease in media attention to H1N1 throughout May 2009. We did not observe the decline in engagement in quarantine measures partly because of the small number of respondents who reported the activities.

Perceived likelihood of infection also varied geographically. Respondents who lived in states with a greater number of H1N1 cases did indeed perceive a higher likelihood of infection, suggesting that respondents were aware of the number of cases in their geographical area. This result was robust when H1N1 per population was used as a measure for geographical risk. Engagement in precautionary activities and interest in pharmaceutical intervention, however, were not found to track this geography-driven difference in perceived risk likelihood.

We also observed a number of demographic differences. Women showed a higher general concern about H1N1 - they perceived higher risk likelihood, were willing to pay more to receive pharmaceutical interventions and more likely to engage in information seeking activities. This gender difference in risk perception is consistent with studies on risk perceptions on health [25,26]. Respondents from larger households undertook more precautionary activities and were more interested in pharmaceutical interventions. Although our demographic data do not allow us to identify the structure of each household, a reasonable guess would be that larger households tend to include a child or children in the household. Influenza transmission from children to adults in a household is often emphasized [27-30], and the positive association between the degree of precautionary behavior and household size would be normatively predicted. In addition, in H1N1 influenza, studies reported that hospitalization rate and mortality caused by infection among children were higher than for seasonal influenza [31,32], which may have further contributed the greater degree of interests and engagement in precautionary behaviors by respondents from larger household. There were few age differences, although older respondents perceived a higher death toll and were willing to pay less to receive pharmaceutical intervention.

We found that perceived likelihood of H1N1 influenza infection tracked objective risk both dynamically and geographically. The temporal dynamic change in risk perception on an infectious disease in response to the objective level of problem was previously found but in a longer time frame [33]. In an emergency situation such as a disease outbreak, however, individuals' risk perceptions could be adjusted in the time frame of days or weeks. In contrast to risk likelihood perceptions that increased over time, respondent's degree of precautionary behaviors declined in willingness to accept preventive intervention and in engagement in information seeking activities, following a pattern similar to the level of media attention. As discussed previously, this decline in precautionary behavior mirrors the decline in media attention. In addition, it also mirrors a sharp decline in Web searched about influenza. *Google Insight^® ^*(Google Inc., Mountain View, CA) indicates that search volume for the search term "flu" showed a tremendous spike right after the outset of the pandemic in April 2009, but quickly returned close to before-pandemic levels in the following two weeks [34]. Data on risk perceptions and behavior change during the initial phase of a disease outbreak are rarely available [13], and our analysis provides useful information on individuals' response associated with the dynamic nature of an infectious disease outbreak.

Our survey results have several implications for successful response to a novel influenza outbreak. Effective vaccination strategies against influenza have been receiving considerable attention [30,35,36]. Successful implementation of an optimal vaccination strategy depends critically on individuals' willingness to accept pharmaceutical and non-pharmaceutical recommendations. As in previous questionnaire research on vaccination behavior [3-5,15,18], willingness to accept a pharmaceutical intervention was associated both with perceived risk and with an individual's adopting precautionary activities. Thus, acceptance of an H1N1 vaccine is likely to be highest among individuals who perceive high risk and who have already engaged in precautionary activities.

Our study also showed that changes over time in willingness to take action tracked temporal changes in media attention. Furthermore, the vast majority of the survey participants reported that they first knew about the H1N1 influenza outbreak through one form of media, suggesting the importance of mass media as an information source. Thus, campaigns to change public behavior may be most successful at the height of media attention, which may occur during the early stages of an outbreak. A previous study showed that national and international public health authorities were the most important source of information on H1N1 influenza in media reports [37]. Therefore a high emphasis should be given to the role of public health authorities in encouraging the public to take the preventive measures.

Finally, individuals in certain demographic categories may be most receptive to pharmaceutical interventions. Young women from large households expressed the highest level of interests in pharmaceutical interventions, and thus may be a potentially successful target of pharmaceutical intervention campaigns. Further study is needed to examine how perceptions and behavior change in response to intervention campaigns.

There are several limitations to our study. First, our sample size was limited and response rate is low. The lows response is partly because our survey was optional and respondents were free to decline any invitation they received. The survey company issued invitations in such a way that the age and gender distribution of participants would approximate that of the US adult population on each day. The survey procedure did not allow us to identify the exact proportion of those who did not participate and of those who were turned away after the daily quota was reached. Second, an optional survey is subject to self-selection bias. In particular, respondents may have been more interested in and concerned about H1N1 influenza than non-respondents, which may result in reporting higher degree of risk perception and/or interest in precautionary behavior than non-respondents. Also our survey was not exempt from limitations that web-based surveys commonly have. For example, gender, age, education background, social status may be related to access to computers or attitude towards web-based surveys [38]. However, most of our results were based on a comparative analysis, which should not be affected by baseline levels of risk perception. Third, we did not collect responses repeatedly from a single cohort but our respondents consist of a different cohort of individuals every day, as in a previous study [13]. Although this collection scheme provided greater sample size to analyze geographical and demographical variations, it resulted in a slightly different distribution on each day of the respondents in terms of sex and age given the limited sample size per day. We therefore controlled for demographic variables in our time-series and geographic analysis. Fourth, our analysis included sex, age, and household size as respondents' characteristics because the primary interest was the dynamics in risk perceptions and precautionary behaviours. Associations with additional respondents' socioeconomic characteristics could be potentially addressed in future research.

## Conclusion

This study tracked individual's risk perceptions and precautionary behavior in an initial stage of a disease outbreak, and provides important insight into temporal and geographical dynamics of risk perception and precautionary behavior. Our result suggested that perceived risk of infection and precautionary behavior were dynamic in time and differed by demographic characteristics and geographical location. These patterns will likely influence the effectiveness of disease control measures.

## Competing interests

The authors declare that they have no competing interests.

## Authors' contributions

All authors participated in the planning of the study and designing of the survey. YI and GBC coordinated the study and took overall responsibility for the delivery of the work, and had responsibility for data collection, data analysis and production of tables and figures. ML had responsibility for data collection. All authors participated in writing the paper, have seen and approved the final version.

## Pre-publication history

The pre-publication history for this paper can be accessed here:

http://www.biomedcentral.com/1471-2334/10/296/prepub

## Supplementary Material

Additional file 1 **Number of respondents by survey day, April 28 - May26 2009**. Table A1 shows the number of respondents who completed the survey on each survey day. Click here for file

Additional file 2 **Respondents by age and sex on each survey day, April 28 - May 26 2009**. Our survey collects a cross-sectional of the US population on each survey day during April 28 and May 26 2009. The distribution of the respondents by age and sex on each survey day is presented in Figure A1. Click here for file

Additional file 3 **Three measures for geographical risk status**. To measure geographical risk status by state, we used: (1) cumulative H1N1 cases in the state; (2) cumulative H1N1 cases per million population; and (3) a dichonomous variable to indicate if one or more deaths were reported in the state as of May 27 2009. (1), (2) and the cumulative number of deaths by state are shown in Table A2. Click here for file
