# Supplementary material for: The dynamics of risk perceptions and precautionary behavior in response to 2009 (H1N1) pandemic influenza
Source: BMC Infect Dis. 2010 Oct 14;10:296. doi: 10.1186/1471-2334-10-296 (PMC2964717; doi:10.1186/1471-2334-10-296)
Supplement: Additional file 1 — Number of respondents by survey day, April 28 - May26 2009. Table A1 shows the number of respondents who completed the survey on each survey day. [file 1471-2334-10-296-S1.pdf]

**Additional file 1 - Number of respondents by survey day, April 28-May26 2009.**

Table A1 shows the number of respondents who completed the survey on each survey day.

*Survey day starts at noon on the day and continues until the target number of respondents initiated the survey.

Table A1: Number of respondents by survey day, April 28-May26 2009

| Survey day * | N |
|---|---|
| April 28 2009 | 442 |
| April 29 2009 | 52 |
| April 30 2009 | 50 |
| May 1 2009 | 44 |
| May 2 2009 | 48 |
| May 3 2009 | 49 |
| May 4 2009 | 47 |
| May 5 2009 | 49 |
| May 6 2009 | 49 |
| May 7 2009 | 47 |
| May 8 2009 | 48 |
| May 9 2009 | 45 |
| May 10 2009 | 44 |
| May 11 2009 | 47 |
| May 12 2009 | 49 |
| May 19 2009 | 91 |
| May 26 2009 | 89 |
