# Supplementary material for: The dynamics of risk perceptions and precautionary behavior in response to 2009 (H1N1) pandemic influenza
Source: BMC Infect Dis. 2010 Oct 14;10:296. doi: 10.1186/1471-2334-10-296 (PMC2964717; doi:10.1186/1471-2334-10-296)

Additional file 2 - Respondents by age and sex on each survey day, April 28- May 26 2009.

Our survey collects a cross-sectional of the US population on each survey day during April 28 and May 26 2009. The distribution of the respondents by age and sex on each survey day is shown in Figure A1.

Figure A1: Respondents by age and sex, on each survey day, April 28 - May 26 2009

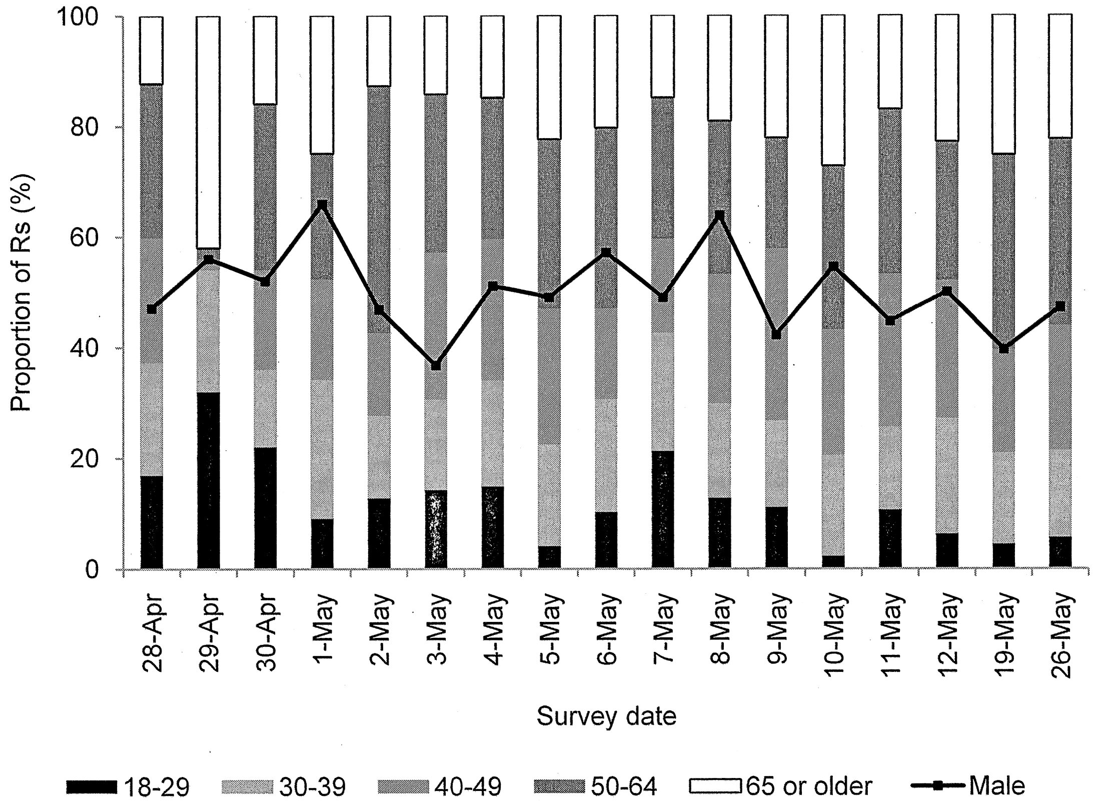

Supplement: Additional file 2 — Respondents by age and sex on each survey day, April 28 - May 26 2009. Our survey collects a cross-sectional of the US population on each survey day during April 28 and May 26 2009. The distribution of the respondents by age and sex on each survey day is presented in Figure A1. [file 1471-2334-10-296-S2.pdf]
