# Supplementary material for: The dynamics of risk perceptions and precautionary behavior in response to 2009 (H1N1) pandemic influenza
Source: BMC Infect Dis. 2010 Oct 14;10:296. doi: 10.1186/1471-2334-10-296 (PMC2964717; doi:10.1186/1471-2334-10-296)
Supplement: Additional file 3 — Three measures for geographical risk status. To measure geographical risk status by state, we used: (1) cumulative H1N1 cases in the state; (2) cumulative H1N1 cases per million population; and (3) a dichonomous variable to indicate if one or more deaths were reported in the state as of May 27 2009. (1), (2) and the cumulative number of deaths by state are shown in Table A2. [file 1471-2334-10-296-S3.pdf]

Additional file 3 - Three measures for geographical risk status

To measure geographical risk status by state as of May 27 2009, we used: (1) cumulative H1N1 cases in the state; (2) cumulative H1N1 cases per million population; and (3) a dichonomous variable to indicate if one or more deaths were reported in the state. (1), (2) and the cumulative number of deaths are shown in Table A2.

Table A2: Three measures for geographical risk status, by state, as of May 27 2009.

State	Cumulative cases*	Cumulative cases per million population*†	Cumulative number of deaths*
Alabama	67	14	0
Alaska	0	0	0
Arizona	532	82	3
Arkansas	12	4	0
California	553	15	0
Colorado	68	14	0
Connecticut	102	29	0
Delaware	108	124	0
Washington, D.C.	14	24	0
Florida	139	8	0
Georgia	28	3	0
Hawaii	44	34	0
Idaho	11	7	0
Illinois	927	72	0
Indiana	120	19	0
Iowa	71	24	0
Kansas	34	12	0
Kentucky	38	9	0
Louisiana	86	19	0
Maine	9	7	0
Maryland	44	8	0
Massachusetts	286	44	0
Michigan	178	18	0
Minnesota	44	8	0
Mississippi	8	3	0
Missouri	24	4	1
Montana	12	12	0
Nebraska	35	20	0
Nevada	50	19	0

New Hampshire	27	21	0
New Jersey	29	3	0
New Mexico	97	49	0
New York	456	23	2
North Carolina	13	1	0
North Dakota	6	9	0
Ohio	15	1	0
Oklahoma	64	18	0
Oregon	120	32	0
Pennsylvania	95	8	0
Rhode Island	11	10	0
South Carolina	39	9	0
South Dakota	4	5	0
Tennessee	95	15	0
Texas	1358	56	3
Utah	122	45	1
Vermont	2	3	0
Virginia	25	3	0
Washington	575	88	1
West Virginia	0	0	0
Wisconsin	1130	201	0
Wyoming	0	0	0

Source: * CDC (2009) [1], †United States Census [2].

References

[1] **Centers for Disease Control and Prevention, 2009 H1N1 Flu**

[http://www.cdc.gov/h1n1flu/]

[2] **United States Census** [http://www.census.gov/]
